# Tamarind Seed Xyloglucans Promote Proliferation and Migration of Human Skin Cells through Internalization via Stimulation of Proproliferative Signal Transduction Pathways

**DOI:** 10.1155/2013/359756

**Published:** 2013-09-09

**Authors:** W. Nie, A. M. Deters

**Affiliations:** Westfalian Wilhelms University of Muenster, Institute for Pharmaceutical Biology and Phytochemistry, Hittorfstraße 56, 48149 Muenster, Germany

## Abstract

Xyloglucans (XGs) of *Tamarindus indica* L. Fabaceae are used as drug vehicles or as ingredients of cosmetics. Two xyloglucans were extracted from *T. indica* seed with cold water (TSw) and copper complex precipitation (TSc). Both were analyzed in regard to composition and influence on cell viability, proliferation, cell cycle progression, migration, MAPK phosphorylation, and gene expression of human skin keratinocytes (NHEK and HaCaT) and fibroblasts (NHDF) *in vitro*. TSw and TSc differed in molecular weight, rhamnose content, and ratios of xylose, arabinose, galactose, and glucose. Both XGs improved keratinocytes and fibroblast proliferation, promoted the cell cycle, and stimulated migration and intracellular enzyme activity of NHDF after endosomal uptake. Only TSw significantly enhanced HaCaT migration and extracellular enzyme activity of NHDF and HaCaT. TSw and TSc predominantly enhanced the phosphorylation of molecules that referred to Erk signaling in NHEK. In NHDF parts of the integrin signaling and SAPK/JNK pathway were affected. Independent of cell type TSw marginally regulated the expression of genes, which referred to membrane proteins, cytoskeleton, cytokine signaling, and ECM as well as to processes of metabolism and transcription. Results show that *T. indica* xyloglucans promote skin regeneration by a direct influence on cell proliferation and migration.

## 1. Introduction

The tamarind is a bushy tree in family Fabaceae and native to tropical Africa. The main components of tamarind seed are xyloglucans. Xyloglucans are one of the two major hemicelluloses in plant primary walls. In general, xyloglucans have a cellulose-like backbone consisting of *β*-1,4-glucosyl residues distributed by short side chains containing fucosyl, arabinosyl, galactosyl, and xylosyl residues [1]. Niemann et al. 1997 [2] characterized the oligosaccharide mixtures after enzymatic digestion of the tamarind seed xyloglucan by endo-(1→4)-*β*-D-glucanase (*Aspergillus niger*) and described that in addition to *β*-D-glucosyl, *α*-D-xylosyl and *β*-D-galactosyl residues some oligosaccharides also contained *α*-L-arabinosyl, and *β*-D-galactosyl-(1→5)-*α*-L-arabinosyl residues. 

In ayurvedic pharmacopeia of India (Part I, Vol IV) the fruits (Ciña) have been noted as laxative. In traditional African medicine the bark and leaves have been often used for treatment of wounds and diarrhea. Tamarind seeds have been powdered and orally administered to treat digestive disorder [2]. Nowadays Tamarind extracts were used for cosmetic preparations [3] and drug vehicles. Tamarind xyloglucans are used in mucoadhesive buccal film [4], microspheres [5], hydrogels [6], and eyedrops [7]. Using tamarind xyloglucans improves percutaneous transport [8], regulates release [9], enhances bioavailability [8], and prolongs systemic absorption [5] of various drugs. Furthermore various investigations were done to lighten the activity of these special xyloglucans on different *in vivo* and *in vitro* models far of the traditional use against digestive disorders. Tamarind seed xyloglucans were shown to reduce UV-induced repression of the delayed-type hypersensitivity response as well as the production of IL-10 in mice [10]. Additionally *in vitro* and *in vivo* studies are available dealing with the antitumor and immune stimulating activity [11, 12], NO production [13], healing properties of dry eye syndrome [14, 15], and cutaneous wounds [16]. Moreover tamarind xyloglucans protected human skin from immune suppression by inhibition of UV-radiation-induced dendritic cell loss from the epidermis [17]. 

The *in vivo* studies underline the effect and benefit of tamarind xyloglucan use in drug delivery systems and in cosmetics the background of the xyloglucans bioactivity has not been investigated so far. *In vitro* cell cultures are an efficient tool for investigation of cell behavior and its causative cellular response. Proliferation and migration are essential for wound regeneration [18] and can be uncomplicatedly investigated *in vitro*. For the present research human skin keratinocytes and fibroblasts were used. These cells construct the dermis and epidermis of skin and are involved in the regeneration and epithelialization of cutaneous wound healing [19]. During the presented study different *in vitro* tests were carried out analyzing the effect of tamarind seed xyloglucans on proliferation, migration, cell viability, cell cycle, and signal pathways in addition to their interaction with cells. Extraction of studies Tamarind xyloglucans was done in different ways. Since variation in precipitation process of water-soluble polysaccharides or using of different solvents and temperatures results in extracts composed of different polysaccharides [20, 21], tamarind seed xyloglucans were extracted by copper precipitation [22] and with cold water to elucidate a possible difference in structure and bioactivity.

## 2. Materials and Methods

Chemicals were purchased in analytical quality from Sigma, Taufkirchen, or Merck, Darmstadt, Germany. Water for cell culture and *in vitro* purpose was purified using a Millipore-System from Millipore, Molsheim, France. If not stated otherwise media and supplements were purchased from PAA Laboratories GmbH (Cölbe, Germany). FCS was supplied by PerBio, Bonn, Germany. Cell culture plates and flasks were obtained from Greiner Bio-One, Frickenhausen, Germany or Sarstedt, Nuembrecht, Germany. MCDB153 medium was obtained from Biochrom, Berlin, and KGM_Gold_ was purchased from Lonza, Cologne, both from Germany. The cells were cultivated at 37°C in an humidified atmosphere at 5% (KGM_Gold_, MEM, and MCDB153 complete) and 8% CO_2_ (DMEM) according to the suppliers instruction.

### 2.1. Isolation of TSw and TSc from *T. indica* Seed

Tamarind Gum (Powder) was kind gift from Professor Dr. Kottapalli Seshagirirao, University of Hyderabad, India. Xyloglucans were isolated according to [22] with a final yield of 27% (w/w) or extracted using cold water as described for Kiwi polysaccharides [23] resulting in 10% water soluble polysaccharide (TSw).

### 2.2. Characterization of TSw and TSc from *T. indica* Seed

The amount of neutral sugars was quantified using resorcinol [24]. Amount of accompanying proteins was measured with Coomassie Brilliant Blue G-250 [25]. The monosaccharide composition was determined by High Performance Anion Exchange Chromatography with Pulsed Amperometric Detection (HPAEC-PAD; Dionex, Idstein, Germany) after hydrolysis with trifluoroacetic acid (TFA, 2 M, at 121°C for 1 h), followed by identification of the D/L configuration by capillary electrophoresis (CE). Performance of HPAEC-PAD and CE referred to [26]. Gel-filtration chromatography (GFC) of TSw and TSc was carried out on a Sepharose CL-6B column (Pharmacia Biotech, Sweden) for determining average molecular weight (MW). Elution was conducted with 0.15 M NaCl (0.3 mL/min). 

### 2.3. Labeling of Tamarind Polysaccharides with Fluorescein Isothiocyanate (FITC)

10 mg of TSw and TSc was labeled with FITC as described by [27]. The labeled polysaccharides were purified by ethanol precipitation (80%) followed by dialyzation against Aqua Millipore (MWCO 3500) and GPC on Sepharose CL-6B. Thin-layer chromatography (TLC) of TSw-FITC and TSc-FITC on silica gel G 60_F254_ was performed to verify the absence of unbound FITC. 

### 2.4. Cell Culture Conditions of NHEK, HaCaT, and NHDF

Normal human dermal fibroblasts (NHDF) were isolated from human skin grafts (Pediatric Surgical Clinic, University of Muenster, Germany) of various Caucasian subjects as described by [23, 28]. The studies were approved by the local ethical committee of the University of Muenster (acceptance number 2006-117-f-S). Adherent spontaneously immortalized HaCaT-keratinocytes were kindly provided by Professor Dr. Fusenig from German Cancer Research Center (DKFZ), Heidelberg, Germany. 

Stock cultures of NHEK grew in KGM_Gold_. Subculture of NHDF was performed in MEM high glucose supplemented with 10% FCS, 1% L-glutamine, and 1% Penicillin/Streptomycin. HaCaT keratinocytes were cultured in D-MEM high glucose (10% FCS, L-glutamine, NEAA, and Penicillin/Streptomycin, each 1%). Cells were allowed to grow to a confluence of 80% before splitting. The studies were performed on the 2nd to 6th (NHDF and NHEK) and 45th–56th passage (HaCaT).

Prior to incubation with xyloglucans cells were directly adapted to serum and BPE-starved media; fibroblasts to MEM high glucose supplemented with 10% SerEx (a defined serum alternate of PAA, Colebe, Germany), 1% L-glutamine, and keratinocytes to MCDB 153 complete medium. Incubation with xyloglucans started 24 h after seeding in serum-starved media when the cells had reached a confluence of 50%. Medium was exchanged against fresh serum-starved medium (untreated control), serum-starved medium containing xyloglucans, and medium supplemented with 10% FCS instead of SerEx (positive control). The incubation was quitted by adding BrdU and MTT reagents after 48 h. Medium was not exchanged during this time. Three independent tests were performed in 96-well plates at starting cell densities of 5 × 10^3^ HaCaT and 3 × 10^3^ NHDF in each well. BrdU incorporation, extracellular enzyme activity (WST-1), and LDH release assays were performed according to the manufacturer's instructions (Roche, Penzberg, Germany). Activity of intracellular reducing enzymes was measured with MTT according to [29]. ATP production of keratinocytes and fibroblasts was quantified using the CellTiter-Glo Luminescent Cell Viability Assay (Promega, USA) and a Fluoroskan Ascent FL (Thermo Scientific, USA).

### 2.5. Determination of DNA Content Using Flow Cytometer for Cell Cycle Analysis

Due to the difference of DNA amount in each cell cycle phase, the cell cycle progression was analyzed by determining the DNA content via flow cytometry. First the human skin cells were seeded in 6-well plates with 1.5 × 10^5^ cells/well (NHEK and HaCaT) and 1 × 10^5^ cells/well (NHDF) and treated with 10 *µ*g/mL TSw or TSc after 3, 6, 12, 24, and 48 h. Afterwards cells were trypsinized, centrifuged, and resuspended in 450 *µ*L PBS. For fixation of the cell status 1.05 mL ethanol (96%, −20°C) was added for 2 h at 4°C. The fixed cells were mixed with 500 *µ*L ice-cold PBS and centrifuged for 10 min at 11000 ×g. The resultant pellet was resuspended in 900 *µ*L PBS followed by the addition of 50 *µ*L RNase solution (1 mg/mL). After incubation in darkness at 37°C for 30 min 50 *µ*L of propidium iodide (PI, 1 mg/mL) were added and stored for further 5 min in darkness. Data were acquired with a FACSCalibur flow cytometer (BD, Heidelberg, Germany). The data were analyzed with FlowJo 7.6.5 software (Treestar Inc., USA).

### 2.6. Scratch Test for Observation of Cell Migration Behavior

Effect of xyloglucans on human skin cell migration was investigated by forming a “wound” in cell monolayer. HaCaTs (3 × 10^4^ cells/well) and NHDF (2 × 10^4^ cells/well) were cultured in 24-well plates to 95% confluence. Growth medium was discarded and cells were washed with PBS buffer. A defined area was scratched with a pipette tip through the monolayer. The detached cells were gently washed away with PBS buffer before 10 *µ*g/mL TSw, respectively, TSc were applied to the cell cultures. 5 *µ*g/mL mitomycin C (MMC) was added to inhibit the closure of the monolayer as a result of proliferation [30]. The scratched area was labeled and photographed with a DFC 300FX camera of a DMIL light microscope (Leica, Mannheim, Germany). The size of the area was calculated using ImageJ software (Wayne Rasband, USA). After 24 h of incubation the scratched area was photographed again and the differences of area size before and after the incubation were computed.

### 2.7. Internalization of TSw-FITC and TSc-FITC into HaCaT and NHDFs

For fluorescence laser-scanning microscopy removable silicone chambers FlexiPERM (Greiner Bio-one, Frickenhausen, Germany) were attached to polylysine-coated glass slides (Menzel GmbH, Braunschweig, Germany) and to slides with ground colored frosted green (VWR, Darmstadt, Germany). 3 × 10^4^ HaCaT were seeded on polylysine-coated slides and 2 × 10^4^ NHDF on the others. At 70% confluence the cells were incubated with 50 *µ*g/mL FITC labeled xyloglucans and 100 *µ*g/mL Dextran-TexasRed (Invitrogen, USA) for 3 h, 6 h, 12 h, and 24 h. Cells were counterstained with 1 *µ*g/mL DAPI 30 min before incubation was finished. Then cells were washed thrice with PBS buffer, fixed with Dako fluorescent mounting medium (Dako, USA) and covered with a cover slip. Fluorescence confocal laser-scanning microscopy was performed on a TCS SP2 fluorescence microscope (Leica, Mannheim, Germany). Internalized FITC-labeled xyloglucans were also quantified with a FACSCalibur flow cytometer using CellQuest Pro software (BD, Heidelberg, Germany). For these investigations HaCaT and NHDF were cultivated in 6-well plates. The rate of internalization of 50 *µ*g/mL of FITC-TSw or FITC-TSc was measured over a timeline of 3 h, 6 h, 12 h, 24 h, and 48 h. On purpose to analyze the amount of internalized xyloglucans TSw and TSc were put on the cells in concentrations of 0.01 *µ*g/mL to 100 *µ*g/mL for 48 h. Before data acquisition cells were trypsinized, washed thrice with PBS, and again resuspended in PBS. Discrimination of internalized from attached xyloglucans was conducted with trypan blue (0,25 mg/mL) that quenches the fluorescence of FITC on cell surface [31].

### 2.8. Gene Expression Analysis

Three independent cultivations of NHEKs and NHDFs were conducted in 6-well plates. Prior to treatment with xyloglucans the cells were directly adapted to MCDB basal containing neither serum, respectively, BPE nor growth factors. After incubation with the 10 *µ*g/mL TSw in serum and growth factor-starved medium for 6 h, the cells were harvested by trypsinization. Isolation and quantification of total RNA, topic-defined PIQOR Skin Microarray, and bioinformatic calculation of 4-fold replicates and statistical analysis were performed by supplier Miltenyi Biotech GmbH, Cologne, Germany. A laser scanner from Agilent (Agilent Technologies, Böblingen, Germany) was used to detect fluorescence signals of the hybridized PIQOR Microarrays. Signal and background intensities of spots were measured using the ImaGene software (Biodiscovery, Hawthorne, CA, USA). Spots were only taken into consideration for calculation of the Cy5/Cy3 ratio that had at least in one channel a signal intensity that was at least 2-fold higher than the mean background. These spots were analyzed with the PIQOR Analyzer software that allows automated data processing of the raw data text files derived from the ImaGene software. In addition, the provided PIQOR Navigator software together with gene cards databank [32] was used for the search for information about the function of a gene of interest. In regard to [33] only genes were accounted with a minimal fold change of 1.5 respective 1.7. 

### 2.9. Analysis of Protein Phosphorylation Profiles via Proteome Profiler Human Phospho-Kinase Array Kit

The Proteome Profiler Human Phospho-Kinase Array kit (R&D Systems, Wiesbaden, Germany) determines the relative phosphorylation level of 46 kinases. After culturing in 25 cm^2^ flasks for 24 h NHEKs and NHDF were treated with 10 *µ*g/mL TSw and TSc for 6 h, harvested by trypsin treatment, and washed with PBS buffer. Cell number was adjusted to 1 × 10^7^ cells that were suspended in 1 mL lysis buffer followed by incubation at 4°C for 30 min with constant shaking. The resultant suspension was centrifuged (11000 ×g, 5 min) and the supernatant was transferred into a clean test tube. The performance and analysis of twice replicated assays from three independent approaches each were done as described by manufacturer. 

### 2.10. Statistics

Statistical analysis was performed by use of SigmaPlot 12 (Systat Software GmbH, Erkrath, Germany). Data were calculated with Holm-Sidak test after analysis of variance (ANOVA). Results are presented as the mean ± standard deviation (SD). Data were considered as statistically significant and highly significant, respectively, with *P* values <0.05 and <0.01.

## 3. Results

### 3.1. Characterization of *T. indica* Seed Xyloglucans

The extraction of TSc, a hot water soluble and copper precipitated xyloglucan, was more complex than the simple extraction of xyloglucan TSw with cold water. It was observed that neither TSw nor Tsc content any protein or uronic acids. As main monosaccharides glucose, xylose, and galactose were determined by HPLC-PAD analysis and capillary electrophoresis. TSw exhibited a seven fold higher molecular weight than TSc and additionally contained slight amounts of rhamnose (Table 1). As well as the arabinose content a closer look revealed that the ratio of glucose and xylose differed. 

### 3.2. Influence of Xyloglucans TSw and TSc on the Cell Viability of Human Skin Cells

**Figure 1:**
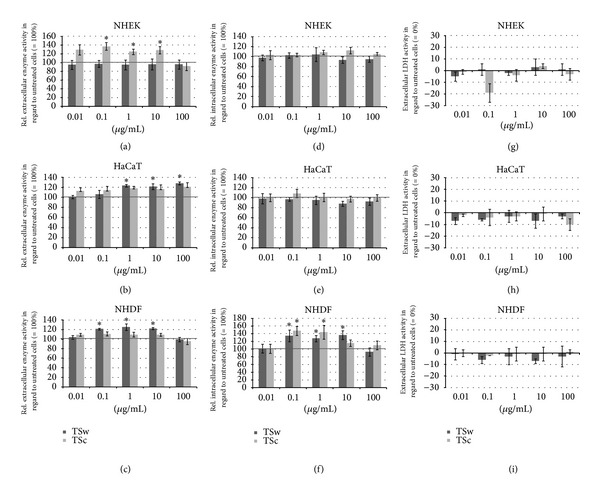
Cell viability of NHEK, HaCaT, and NHDF after incubation with 0.01–100 *µ*g/mL TSw and TSc for 48 h normalized to activity of untreated control cells (black line). Extracellular enzyme activity measured by WST-1 reduction is shown in (a) (NHEK), (b) (HaCaT), and (c) (NHDF). MTT reduction as indicator for intracellular enzyme activity is presented in (d) (NHEK), (e) (HaCaT), and (f) (NHDF). (g), (h), and (i) depict the extracellular LDH activity compared to extracellular LDH activity of untreated cells (=0%). Presented are the mean values ± SD of *n* = 8 replicates of three independent experiments with *P < 0.05 compared to untreated cells.

The tetrazolium salts MTT and WST-1 were chosen to investigate the activity of reducing enzymes in the cells and as parts of the cell membrane [34]. TSw did not affect the intra- and extracellular metabolic activities of NHEK (Figures 1(a) and 1(d)) whereas the activity of extracellular reducing enzymes of HaCaT keratinocytes was significantly enhanced (Figure 1(b)). Though TSw showed no influence on MTT reduction by HaCaT cells (Figure 1(e)). Contrary to keratinocytes, 0.1 *µ*g/mL–10 *µ*g/mL of TSw significantly increased the extra- and intracellular enzyme activities of NDHF (Figures 1(c) and 1(f)).

0.1 *µ*g/mL, 1 *µ*g/mL, and 10 *µ*g/mL TSc significantly improved the extracellular enzyme activity of NHEK (Figure 1(a)) and intracellular reduction of MTT by NHDF (Figure 1(f)). The extracellular enzyme activity of HaCaT was also enhanced but not significantly compared to untreated cells. Intracellular enzyme activity of keratinocytes was not affected. For elucidation of direct cytotoxic activity the extracellular LDH activity in cell cultures incubated with tamarind seed xyloglucans was measured. After 48 h of incubation neither TSw nor TSc exhibited no cytotoxic effects in concentrations of 0.01–100 *µ*g/mL. Quite the contrary compared to untreated control cells the amount of extracellular LDH was slightly reduced (Figures 1(g), 1(h), and 1(i)). 

Adenosine-5′-triphosphate (ATP) is the molecular unit to save chemical energy. Energy transfer during the metabolism is characterized by conversion of ATP to its precursors and a turnover back to ATP. The level of ATP determines the energy status of a cell. The energy status of normal skin cells was not influenced after incubation with tamarind seed xyloglucans. However 10 *µ*g/mL TSw and TSc slightly increased the ATP amount of immortalized HaCaT keratinocytes in regard to untreated cells (100%) as shown in Table 2. 

### 3.3. Cell Proliferation after Incubation with Tamarind Seed Xyloglucans

**Figure 2:**
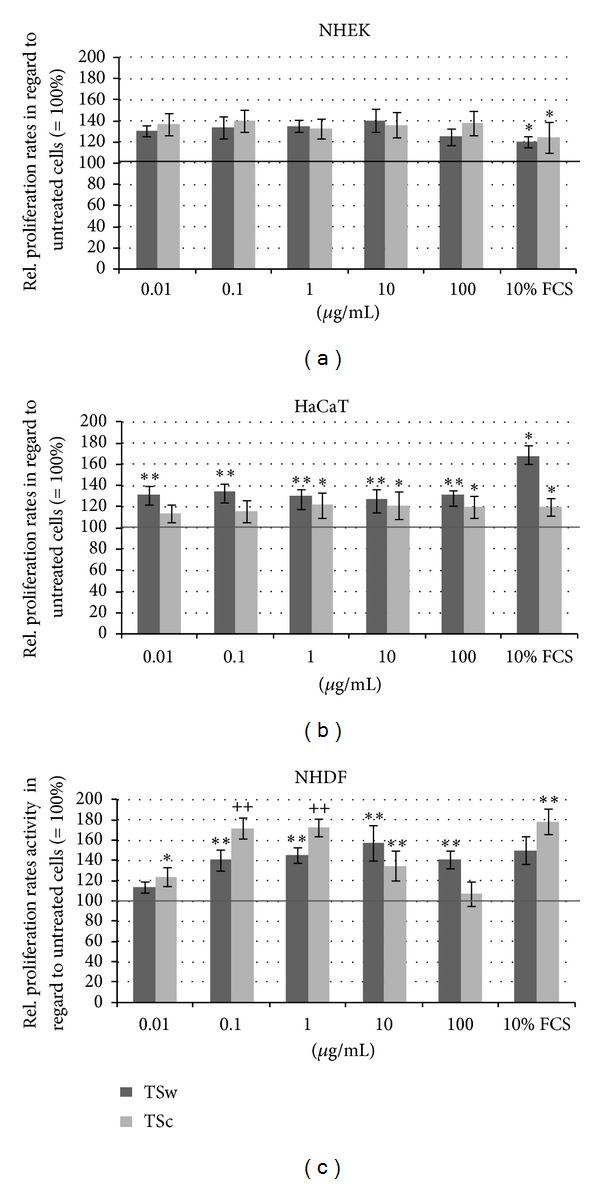
BrdU incorporation as marker for cell proliferation. NHEK (a), HaCaT (b), and NHDF (c) were incubated with TSw and TSc in concentrations of 0.01–100 *µ*g/mL for 48 h. Mean values ± SD of *n* = 8 replicates of three independent experiments are shown with *P < 0.05 and **P < 0.01 compared to untreated cells as well as with ^++^ *P* < 0.01 compared to cells treated with 0.01 *µ*g/mL and 10 *µ*g/mL TSc; 10% FCS = positive control. Results were normalized to untreated cells (=100%, black line).

The proliferation of NHEK highly significantly increased in a dose-independent manner if TSw was applied with increasing concentration in a range of 0.01–10 *µ*g/mL. 100 *µ*g/mL TSw reduced the NHEK proliferation rates but the differences were not significant (Figure 2(a)). TSw significantly elevated the proliferation of HaCaT keratinocytes but independent of the used dosage (Figure 2(b)). As presented in Figure 2(c) TSw also boosted the proliferation of normal fibroblasts but not dependent of the used concentration. In comparison to NHEK the effect started not with a concentration of 0.01 *µ*g/mL TSw but with 0.1 *µ*g/mL (Figure 2(c)).

Similar to TSw the copper precipitated xyloglucan TSc increased the cell proliferation of fibroblasts and keratinocytes. No dependence on used concentration was observed with NHEK (Figure 2(a)). HaCaT cell proliferation was also enhanced with a visible but not significant step between concentrations below and above 1 *µ*g/mL (Figure 2(b)). TSc mostly effected NHDF proliferation in dosages of 0.1 and 1 *µ*g/mL with a 70% increase of proliferation rates. This effect was significant compared to the results obtained with 0.01, 10, and 100 *µ*g/mL (Figure 2(c)).

In addition the particular cell cycle phases were analyzed when cells were incubated with TSw and TSc. Compared to control cells both xyloglucans triggered keratinocytes and fibroblasts to switch from G_0_/G_1_ into S- and, respectively, or G_2_/M-phases.

**Figure 3:**
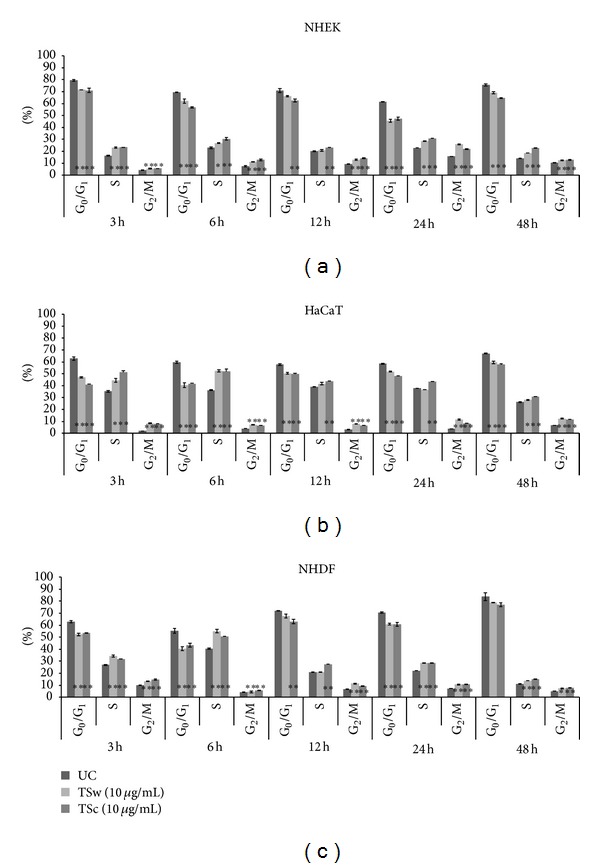
Flow cytometric analysis of cell cycle progression. The cells NHEK (a), HaCaT (b), and NHDF (c) were incubated with TSw, respectively, TSc from 3 h to 48 h. The percentage of cells in entire cell cycle was considered as 100%, presented are mean values ± SD with *n* = 2 replicates of two independent experiments; *P < 0.05, **P < 0.01 compared to untreated cells. UC: untreated cells.

The number of NHEK in S-phase increased 30% after 24 h when they had been incubated with both xyloglucans. Also more than 20% of NEHK were in G_2_/M-phase at this time. After 6 h, 12 h and 48 h the number of xyloglucan-treated NHEK in G_2_/M-phase was twofold enhanced compared to untreated NHEK while the number of NHEK in S-phase remained nearly the same (Figure 3(a)). When TSw and TSc were applied to HaCaT keratinocytes the number of cells in S-phase rose during the first 6 h to a maximum of 53% (TSw), respectively, 52% (TSc) and decreased to a minimum of 28% at 48 h. The amount of cells in G_2_/M rose when HaCaT was incubated with TSc and TSw, too, but differences did not exceed 15%. The maximum values of HaCaT in G_2_/M-phase were obtained 48 h after incubation. The number of HaCaTs in S- and G_2_/M-phase was elevated compared to untreated cells every time (Figure 3(b)). NHDF incubated with TSw and TSc for 3 h showed an increase of cells in S-phase and a maximum of cells in G_2_/M-phase compared to untreated cells. The most NHDF in S-phase was observed 6 h after incubation of TSw (55%) and TSc (51%). Further incubation resulted in a reduced number of NHDF in S-phase and G_2_/M-phase but remained enhanced compared to untreated NHDF (Figure 3(c)). 

### 3.4. Effect of Tamarind Seed Xyloglucans on the Migration of Skin Cells

Migration analysis of NHEK was not possible when mitomycin C (MMC) was used because NHEK started to differentiate. Even though the use of different media resulted in a differentiation of NHEK. This effect was not seen with HaCaT cells because they lost the ability to differentiate. 

Compared to untreated control and to positive control, the cold water soluble tamarind xyloglucan TSw improved the closure of the scratched area of a HaCaT monolayer. TSc-treated HaCaT-scratched monolayer was not closed and the scratched area was as large as in the culture of untreated cells. The monolayer of scratched NHDF was closed during 24 h when TSc, TSw, or 10% FCS were applied. The tamarind xyloglucans were as active as 10% FCS (Table 3). 

### 3.5. Internalization of TSw and TSc in Human Keratinocytes and Fibroblasts

Previous presented results showed slight differences in composition and in bioactivity of tamarind seed xyloglucans TSw and TSc. Due to the immense difference in molecular weight the question rose according to the way of activity especially of an activity from outside or inside the cells. 

**Figure 4:**
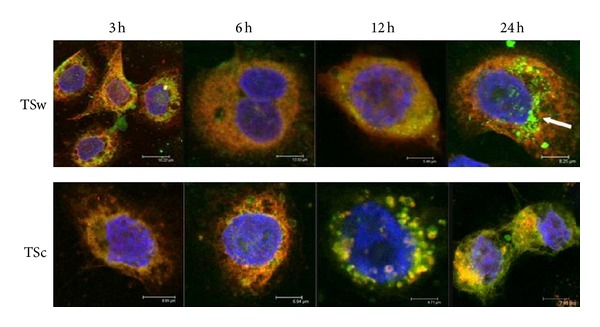
Laser-scanning confocal microscopy for internalization of green fluorescent FITC-TSw, respectively, FITC-TSc (50 *µ*g/mL) in HaCaT after incubation of 3 h, 6 h, 12 h, and 24 h. Nuclei were labeled with blue fluorescent DAPI (1 *µ*g/mL). Green spots: FITC-TSw, respectively, FITC-TSc; red spots: Dextran-TexasRed labeled endosomes; yellow spots: Colocalization of FITC-TSw, respectively, FITC-TSc and Dextran-TexasRed labeled endosomes structures. Arrows point to zones of not internalized FITC-labeled xyloglucans.

The uptake of TSw in HaCaT was already observed after incubation of 3 h. Until 12 h most FITC-labeled TSw was localized in endosomes which were spread over the whole cell. During further incubation green spots got obvious at the periphery of the nucleus (Figure 4). Quantification by flow cytometry revealed that the endosomal uptake of FITC-TSw reached a maximum at 24 h and decreased during further 24 h as observed by focal laser-scanning microscopy (Figure 6(a)). The overlay of fluorescence images FITC-TSc-treated cells presented an increasing yellow fluorescence 3 h after addition to HaCaT with a maximum at 24 h (Figure 4). Quantification of yellow fluorescence resulted in a stepwise increase until 12 h incubation. When the incubation was prolonged the intensity resisted (Figure 6(b)). 

**Figure 5:**
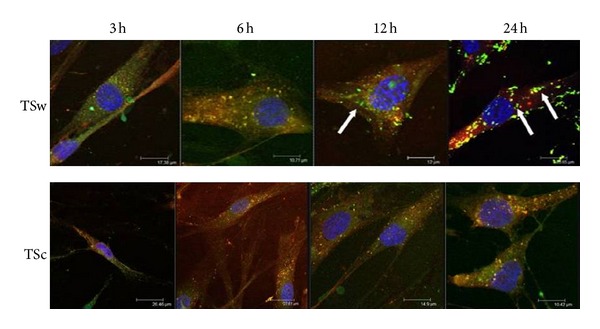
Internalization of green fluorescent FITC-TSw, respectively, FITC-TSc (50 *µ*g/mL) by NHDF analyzed via laser-scanning confocal microscopy after incubation of 3 h, 6 h, 12 h, and 24 h. Blue fluorescence: DAPI-stained nuclei; red fluorescence: endosomes labeled with Dextran-TexasRed; yellow fluorescence: colocalization of FITC-TSw, respectively, FITC-TSc and Dextran-TexasRed within the endosomes.

**Figure 6:**
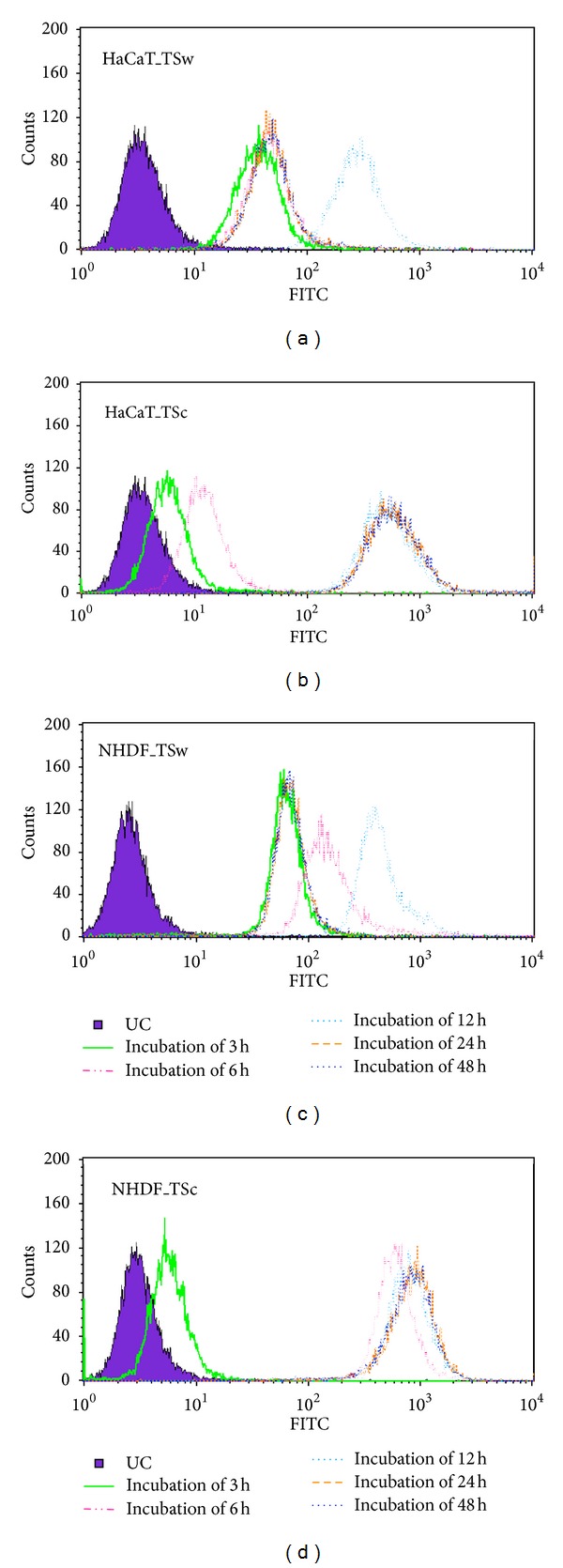
Fluorescence intensity of 50 *µ*g/mL FITC-TSw ((a) and (c)) and FITC-TSc ((b) and (d)) in HaCaT and NHDF measured with flow cytometer after incubation for 3 h to 48 h.

The images of NHDF after treatment with FITC-labeled xyloglucans revealed that FITC-TSw was internalized by endosomes between 3 h and 6 h. After 12 h and 24 h the yellow-colored spots (representing FITC-TSw loaded endosomes) condensed and were spread all over the cell (Figure 5). Additionally an increasing number of red- and green-labeled spots were detected at these times. The decrease of FITC-TSw loaded endosomes and the increase of unloaded endosomes as well as free green fluorescence were measured during flow cytometry after incubation for 24 h and 48 h (Figure 6(c)). Until 12 h a stepwise increase of yellow fluorescence intensity was observed. In case of FITC-TSc an increase of yellow fluorescence was already shown after 3 h. During 24 h the internalization increased and resulted in a condensation of FITC-TSc loaded endosomes at the periphery of the nucleus (Figure 5). But flow cytometric analysis revealed that the maximum internalization was reached not before 6 h. The intensity of yellow fluorescence increased only to less extends at an incubation time of 24 h. This intensity level did not change during further 24 h (Figure 6(d)).

**Figure 7:**
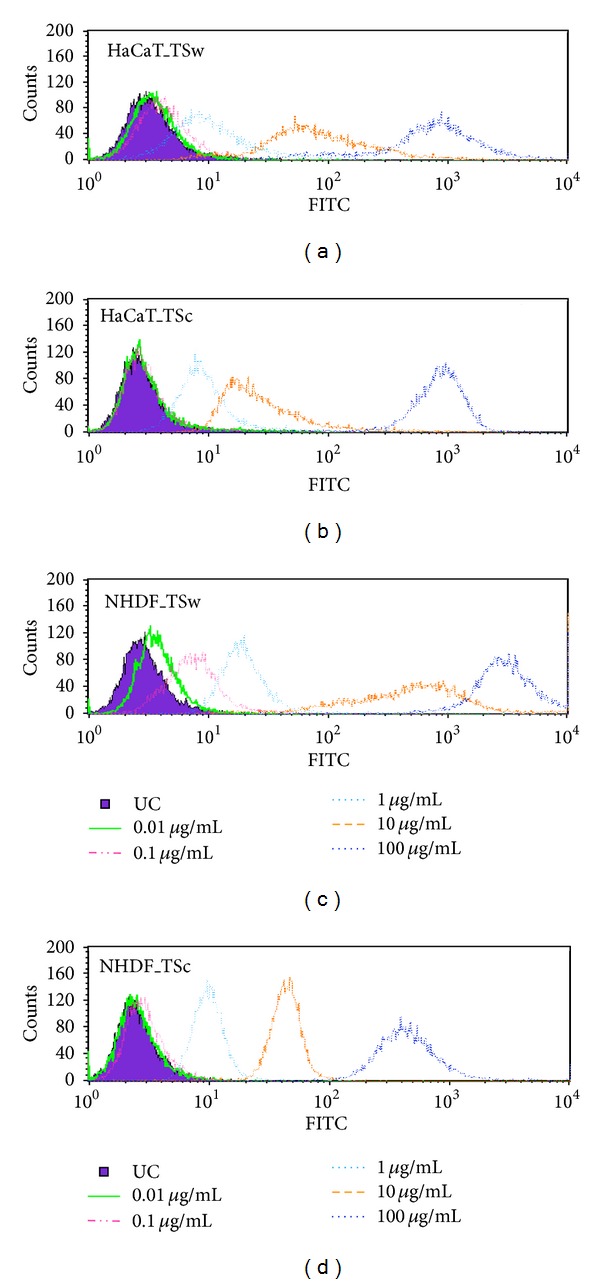
Fluorescence intensity of FITC-TSw ((a) and (c)) and FITC-TSc ((b) and (d)) applied to HaCaT ((a) and (b)) and NHDF ((c) and (d)) in concentrations of 0.01 *µ*g/mL–100 *µ*g/mL. Incubation took place for 48 h.

The investigations concerning cell viability, proliferation, and migration revealed no significant differences in activity in relation to the used concentration. Furthermore no inhibition or homeostasis of cell physiologic activities was observed when 100 mg/mL xyloglucan was added. For that the capability of cells to internalize different amounts of xyloglucans was studied with FITC-labeled xyloglucans in a flow cytometric assay. As depicted in Figure 7 an increase of fluorescence intensity was already seen after incubation of 0.01 mg/mL FITC-TSw in HaCaT (A) and NHDF (C). The relationship of fluorescence intensity of internalized FITC-TSw and applied concentration was linear with a coefficient of determination *R* ^2^ > 0.999 (HaCaT) and *R* ^2^ > 0.997 (NHDF). In case of FITC-TSc an increase of fluorescence intensity was observed after incubation with 0.1 *µ*g/mL (NHDF), respectively, 1 *µ*g/mL. The linearity of uptake was determined with *R* ^2^ > 0.998 (HaCaT) and *R* ^2^ > 0.999 (NHDF). The results exhibit that the capacity of skin cells to internalize xyloglucans did not depend on the molecular weight of xyloglucans and the capacity is not exhausted with 100 *µ*g/mL.

### 3.6. Phosphorylation of MAPK after Incubation with TSw and TSc

The effect of Tsw and Tsc on the phosphorylation status of different proteins involved in cell signaling was measured after incubation for 6 h. The proteins are mitogen-activated protein kinases (MAPK) as well as their protein substrates that refer to ERK signaling, SAPK/JNK pathway, Wnt/*β*-Catenin signaling, and Akt signaling.

Neither NHEK nor NHDF showed a significant inhibition of phosphorylation of the tested proteins 6 h after incubation with TSw and TSc. No influence was observed on the phosphorylation of p53, GSK-3*α*/*β*, Chk-2, Lck, Paxillin, p27, STAT4, Akt_T308_, and AMPK*α*1,2. In response to TSw or TSc MEK1/1, PLC-*γ*, CREB, JNKpan, c-Jun, p70S6_T229_ and p70S6_T289_, eNOS, Fyn, Lyn, Pyk-2, and STAT4 were phosphorylated in NHEK but not in NHDF. On the other hand Akt_S473_, FAK, Fgr, Hck, Yes, STAT5b, and STAT6 were activated in NHDF and not in NHEK. ERK1/2, RSK1/2, p38*α*, *β*-Catenin, and STAT5 were phosphorylated independent of the used xyloglucan and the respective cell type. As shown in Table 4 the incubation of skin cells with TSc resulted in a promotion of the phosphorylation of STAT upstream proteins Fyn, Lyn, Src, and Yes and additionally of the most STATs including STAT1, 2, 3, 5a, and 5a/b in NHEK and NHDF. Furthermore TOR and of p70S6_T421_ (TSw) as well as Akt_S473_ (TSc) were activated in NHDF. With exception of MSK1/2 and JNKpan both tamarind xyloglucans generally induced the phosphorylation of proteins involved in MAPK cascades especially in NHEK. Whereby, incubation with TSw resulted in a predominant phosphorylation of downstream MAPK.

### 3.7. Gene Expression of Skin Cells after Incubation with Tamarind Xyloglucans

**Figure 8:**
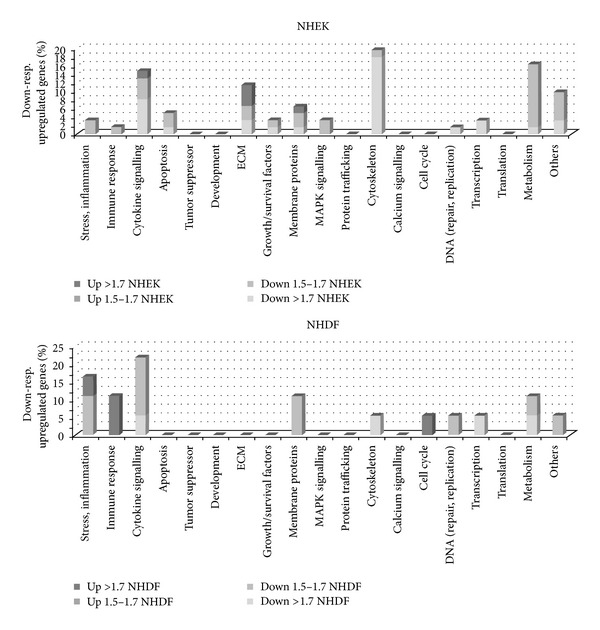
Genes regulated in NHEK, respectively, NHDF determined with the topic-defined PIQOR skin microarray after incubation of cells with 10 *µ*g/mL TSw for 6 h. Presented is the ratio of regulated genes in percent in regard to the total number of regulated genes. The amount of not regulated genes is not presented. The minimal fold change is significant if the ratio is >1.7.

Regulation of gene expression occur in response to extra- or intracellular signals. Close relation had been shown between extra- or intracellular signals and pathways which lead the signals through the cell. In regard to the speed of signal transduction the gene expression mostly starts between 3 h and 6 h, prolongs to 12 h, and decreases after 24 h. Evaluation of NHDF and NHEK gene expression after treatment with 10 *µ*g/mL TSw in serum and growth factor-starved medium for 6 h showed that only 5% (NHEK) and 2% (NHDF) of all 1303 investigated genes were regulated. Furthermore mostly gene expression was inhibited. As presented in Figure 8 the regulated genes referred to apoptosis-related pathways, extracellular matrix (ECM) proteins, growth factors, MAPK signaling, cytoskeleton, metabolism, cytokine signaling, and membrane proteins in NHEK. Genes related to cytokine signaling, stress or inflammation, immune response, cell cycle, membrane proteins, and metabolism were affected in NHDF. The expression of tumor suppressors, as well as genes of development, protein trafficking, calcium signaling, and translation was not seen as response to the incubation with TSw at this time. 

## 4. Discussion

The intention of the presented study was to investigate the effects of xyloglucans from *T. indica* seeds on cell viability, proliferation, and migration of human skin cells and to study the response in regard to molecular changes in signal transduction and endocytosis. 

Investigations were carried out with two xyloglucans differing in molecular weight and composition as cause of extraction method. The diversity of tamarind xyloglucans has been described in a variety of publications [35]. The main sugar composition of TSw with L-arabinose, D-galactose, D-xylose, and D-glucose in ratios of 0.46 : 1 : 2.64 : 2.43 was in line with the report from [36] which also purified the tamarind xyloglucan with water extraction. This xyloglucan was identified as a galactoxyloglucan containing (1→4)-*β*-D-glucan backbone branched by *α*-D-xylopyranose and *β*-D-galactopyranosyl-(1→2)-*α*-D-xylopyranose through (1→)-link; additionally a minority of this xyloglucan possessed not branched (1→4)-beta-D-galactopyranan and branched (1→5)-alpha-L-arabinofuranan residues. The monosaccharide composition of TSc (arabinose : galactose : xylose : glucose 0.16 : 1 : 2.23 : 4.7), which was extracted according to [22], went conform with the xyloglucan that was described by ibid and [2]. 

The bioactivity of the isolated xyloglucans TSw and TSc resembled at the first sight. Both significantly improved the cell proliferation of keratinocytes and fibroblasts as well as the migration of NHDF. Neither the cold water extracted TSw nor the through copper precipitation isolated TSc offered cytotoxic effects. Both xyloglucans were internalized by endosomes in NHDF and HaCaT and promoted the cell cycle progression. But differences were observed in regard to cell viability, migration of HaCaT, internalization time, signal transduction, and gene expression. 

Within 3 h of incubation TSw was rapidly internalized by keratinocytes and fibroblasts. Cell cycle analysis revealed that the TSw-treated cells passed on to S-phase already after 6 h and again after 24 h and 48 h of incubation compared to untreated control cells. The increase of cells in S-phase after 48 explained the results of BrdU test. In both cell types the downstream kinases as well as the transcription factors were phosphorylated. The receptor-related kinases and the other upstream kinases were not activated with exception of PLC-*γ* in NHEK, Src in NHDF, and *β*-catenin in NHDF and NHEK. Kinases like p53 or GSK-3*α*/*β* that regulate cellular homeostasis were not affected. The most phosphorylated kinases participate in the Erk pathway. Difference in phosphorylation of MAPK was observed concerning STAT6, STAT2, and Src that were activated in NHDF and PLC-*γ*, CREB, c-Jun, eNOS, and p70 S6 at T229, respectively, T389 that were solely phosphorylated in NHEK. Phosphorylation of TOR, p70 S6_T229_, and p70S6_T389_ in NHEK as well as p70S6_T421_ and STAT3 in NHDF displayed that contiguous to the Erk signaling different pathways were affected. The protein complex TOR regulates cell survival and growth by activation of p70S6 [37] or STAT3 [38]. Recent publications showed that the STAT6 pathway is involved in nutrient metabolism and regulates insulin sensitivity [39]. STAT2 is commonly involved in IFN-mediated immune response while STAT1 and STAT5 are known to be activated by different growth factors forming dimers STAT2 [32]. Although the startpoint is not clarified, TSw increased phosphorylation of signal molecules of pathways leading to cell survival, proliferation, and migration. The underlying signaling pathway could not be identified owing to the less regulation of genes after 6 h of incubation. Compared to other polysaccharides only a few genes were expressed due to the incubation with TSw [40]. As signal transduction was ongoing, shown by MAPK phosphorylation, regulation of gene expression was marginally started and must be evaluated after longer incubation times. At this early stage gene expression mostly concerned genes that refer to proteins of ECM, cytokine signaling, membrane proteins, and cytoskeleton in NHEK whereas expression of genes involved in stress response or and cytokine signaling were induced in NHDF. Nevertheless MAPK phosphorylation and gene expression results show that the different effect of TSw on cell viability and migration of fibroblasts and keratinocytes depends on a modified signal transduction.

As TSw the copper-precipitated xyloglucan TSc improved skin cell proliferation but only promoted the migration of NHDF. On the other hand TSc was more effective on extracellular reducing enzymes of NHEK and on intracellular reducing enzymes in NHDF. Interestingly, despite the lower molecular weight TSc internalization was prolonged compared to TSw as it required 6 h in keratinocytes and almost 12 h in NHDF. Furthermore the phosphorylation analysis showed that the signal transduction was less advanced in cells that were incubated with TSc. In detail upstream kinases of Src family (FAK, Fgr, Fyn, Hck, and Yes) as well as the STATs, which are commonly activated close to the cell membrane, were phosphorylated in NHEK and NHDF to a higher extent. Differences were observed when cell type was contemplated. Only Fyn was phosphorylated in both cell types whereas TSc mostly activated Fak, Fgr, and Hck in NHDF but not in NHEK. The phosphorylation of down-stream MAPK was nearly independent of used xyloglucan in NHEK. But the phosphorylation of c-Jun and JNKpan as well as the inhibition of STAT3 phosphorylation suggests that TSc affected the SAPK/JNK signal cascade additionally to Erk signaling in NHEK [40, 41]. Similar to TSw the growth inhibitory kinases were not phosphorylated in cells incubated with TSc.

An interesting fact was that both xyloglucans with their high molecular weight were internalized by the cells and that the internalization of the huge TSw (437 kDa) was faster than the uptake of TSc (63 kDa). So a high molecular weight did not anticipate the uptake. The yellow color, a result of overlay of Texas Red Dextran labeled endosomes and FITC labeled xyloglucans, reveals that the uptake was done by endosomes. Fluorescence microscopic analysis demonstrated that the colocalization of FITC-TSw and endosomes was abrogated within 12 h (NHDF) and 24 h (HaCaT). So would they be metabolized and used as nutrients and then stimulated the cell proliferation or did they directly affect the signal transduction? The first part of the question must be cleared in further experiments. But results of MAPK phosphorylation might explain the second part. While TSc and TSw were internalized by endosomes, the phosphorylation of MAPK was altered compared to untreated cells. For that it is possible that the signal transduction was influenced through the xyloglucan uptake as it had been described for clathrin-mediated and clathrin-independent endocytosis [42]. The phosphorylation of Lyn, Fyn, Hck, and protein tyrosine kinases (PTK) pointed to a non-clathrin-mediated endocytosis [43] but that has to be investigated in future. That the character of cargo predetermines the endocytotic uptake and the influence on signal transduction [44] might explain the differences in the influence on MAPK phosphorylation. Analysis on MAPK phosphorylation and gene expression could not clearly identify the signal pathways leading to an increase of proliferation and migration of skin cells incubated with xyloglucan. But the presented results limited the number of possible pathways. Reactions to irritation and inflammation are very fast and had been obvious during 6 h on gene expression level. Apoptosis-inducing pathways seemed to be inhibited and cell proliferation inhibiting pathways were affected neither on gene nor on protein or cellular level. Depending on results of MAPK phosphorylation the integrin [45], SAPK/JNK [40, 41, 46], and ERK signaling [40] could be involved in reaction of cells to xyloglucans. The activation of proproliferative pathways and signal molecules known to be involved in cancer might be aware of using Tamarind xyloglucans for wound healing, drug vehicle, digestive disorders, or dry eye syndrome. But concern is appeased with moderate activation of these molecules *in vitro* as well as the results of various *in vivo* studies that proofed the lack of carcinogenicity [11, 47] and the benefit for wound healing [16].

## 5. Conclusion 

TSw and TSc differed immensely in molecular weight but slightly in composition. So it remains open if single monosaccharides of side chains or the molecular weight and overall structure were responsible for differences in internalization and bioactivity. The results showed that the bioactivity of a xyloglucan depends on its extraction and on the cell type that would be affected. Nevertheless, the effectivity of tamarind xyloglucans for wound-healing properties was proved because they improved processes of reepitheliaization and remodeling. In concern of xyloglucan's use in different drug formulations it should be taken in mind that they are not only an inactive excipient but also might affect the cells at the application site even if this effect is beneficial for the cells.

## Figures and Tables

**Table 1:** Monosaccharide composition of TSw and TSc from *T. indica* seeds determined by HPAEC-PAD and CE after total hydrolysis with TFA (%, w/w). The average MW of TSw and TSc was determined by GFC against dextrans of known molecular weight on Sepharose CL-6B.

	Cold water soluble TSw	Copper precipitated TSc
L rhamnose	2.0	0
L arabinose	6.9	1.9
D galactose	15.0	12.2
D glucose	36.4	57.4
D xylose	39.6	28.5

Average MW [kDa]	437	63

**Table 2:** Energy status of NHEK, HaCaT keratinocytes, and NHDF after incubation with 10 *μ*g/mL Tamarind xyloglucans TSw and TSc. Shown are mean values ± SD of *n* = 8 replicates of two independent experiments.

TSw			TSc		
NHEK	HaCaT	NHDF	NHEK	HaCaT	NHDF
91.78 ± 11.22	124.59 ± 14.56	98.96 ± 14.28	101.86 ± 12.37	108.33 ± 2.72	89.28 ± 8.08

**Table 3:** Effect of tamarind xyloglucans on migration of HaCaT and NHDF. Migration was investigated by scratch assay for 24 h.

	TSw (10 *μ*g/mL)	TSc (10 *μ*g/mL)	PC (10% FCS)
HaCaT	310 ± 9%**	104 ± 20%	197 ± 5%**
NHDF	147 ± 12%**	143 ± 7%**	144 ± 17%**

PC: positive control with 10% FCS. Values are mean ± SD related to untreated cells (=100%) of *n* = 4 replicates of three independent experiments, *P < 0.05, **P < 0.01 compared to untreated cells.

**Table 4:** Relative protein phosphorylation levels related to untreated cells after treatment of NHEK and NHDF with 10 mg/mL TSw and TSc for 6 h measured with the Proteome Profiler Human Phospho-Kinase Array kit. An increase or decrease of >20% was considered as significant.

MAPK	NHEK		NHDF	
	TSw	TSc	TSw	TSc
Upstream MAPK				
*β*-Catenin	**133,06** ± 1,20	**126,01** ± 2,23	**141,65** ± 1,13	**131,62** ± 2,38
FAK	104,81 ± 0,24	110,06 ± 2,59	100,55 ± 1,28	**127,12** ± 0,93
Fgr	105,95 ± 0,06	111,22 ± 3,05	98,48 ± 0,56	**127,24** ± 1,56
Fyn	102,18 ± 0,63	**121,00** ± 0,14	99,01 ± 0,53	104,46 ± 0,14
Hck	103,86 ± 0,05	105,10 ± 0,99	119,15 ± 1,06	**125,04** ± 0,61
Lyn	109,22 ± 0,70	**124,27** ± 1,02	118,27 ± 0,19	110,09 ± 0,25
Yes	110,89 ± 0,11	119,43 ± 1,23	99,64 ± 0,10	**128,49** ± 0,35
Src	108,57 ± 2,92	**123,20** ± 4,24	**120,78** ± 0,20	**129,15** ± 0,74
PLC*γ*-1	**128,85** ± 2,99	**131,00** ± 2,81	110,24 ± 0,48	102,65 ± 1,22
Downstream MAPK				
MEK1/2	**123,56** ± 2,57	**125,09** ± 1,56	101,89 ± 0,97	106,50 ± 4,19
ERK1/2	**135,51** ± 2,21	**138,77** ± 0,16	**140,68** ± 1,32	**174,00** ± 0,20
p38*α*	**136,46** ± 0,66	**123,92** ± 1,74	**121,99** ± 0,27	**123,37** ± 0,64
TOR	**124,83** ± 1,42	**124,38** ± 1,22	**121,25** ± 0,11	113,47 ± 0,27
Akt_S473_	109,47 ± 0,23	114,84 ± 0,76	120,33 ± 0,95	**121,18** ± 0,21
HSP27	**125,22** ± 0,20	**123,86** ± 0,98	**129,28** ± 0,77	111,02 ± 2,82
MSK1/2	**158,29** ± 1,07	**147,02** ± 0,32	**129,84** ± 0,64	114,41 ± 2,08
RSK1/2	**124,35** ± 0,23	**129,07** ± 0,81	**121,93** ± 0,27	108,05 ± 5,76
RSK1/2/3	**123,08** ± 0,40	**129,31** ± 0,56	**127,61** ± 0,59	**124,46** ± 0,59
p70 S6_T229_	**122,07** ± 0,77	**133,79** ± 2,27	109,13 ± 0,80	107,49 ± 0,31
p70 S6_T389_	**138,98** ± 0,72	**143,35** ± 6,93	105,90 ± 1,09	100,43 ± 0,52
p70 S6_T421_	**123,33** ± 0,19	**130,05** ± 0,16	**126,00** ± 0,75	117,22 ± 0,01
JNKpan	118,99 ± 1,41	**154,76** ± 0,24	102,72 ± 2,12	103,13 ± 1,48
eNOS	**123,01** ± 0,33	**124,12** ± 3,02	116,37 ± 0,53	103,72 ± 0,67
Transcription				
c-Jun	**125,84** ± 0,39	**126,25** ± 0,12	104,08 ± 0,10	92,42 ± 0,85
CREB	**134,43** ± 0,40	**131,86** ± 0,62	98,90 ± 2,15	102,68 ± 2,10
STAT1	**130,79** ± 0,36	**132,63** ± 0,55	**126,87** ± 2,15	111,37 ± 0,92
STAT2	118,20 ± 0,90	**122,22** ± 0,22	**123,78** ± 0,51	**126,55** ± 0,61
STAT3	**121,22** ± 0,86	115,62 ± 2,41	**121,89** ± 0,18	**124,50** ± 0,06
STAT5a	**126,52** ± 0,90	**125,32** ± 2,02	**120,40** ± 0,03	**120,60** ± 0,21
STAT5a/b	**121,46** ± 0,86	118,70 ± 0,13	**121,63** ± 0,27	**120,78** ± 0,24
STAT5b	109,81 ± 0,78	104,03 ± 0,68	100,65 ± 1,32	**123,94** ± 0,26
STAT6	105,77 ± 0,22	107,15 ± 3,14	**137,50** ± 0,38	**132,08** ± 7,89

## References

[1] Fry SC (1989). The structure and functions of xyloglucan. *Journal of Experimental Botany*.

[2] Niemann C, Carpita NC, Whistler RL (1997). Arabinose-containing oligosaccharides from tamarind xyloglucan. *Starch/Staerke*.

[3] Abraham Tholath E, Ghandroth KS Transparent Xyloglucan/Chitosan Gel and a Process for the Preparation Thereof.

[4] Avachat AM, Gujar KN, Wagh KV (2013). Development and evaluation of tamarind seed xyloglucan-based mucoadhesive buccal films of rizatriptan benzoate. *Carbohydrate Polymers*.

[5] Pal D, Nayak AK (2012). Novel tamarind seed polysaccharide-alginate mucoadhesive microspheres for oral gliclazide delivery: *in vitro-in vivo* evaluation. *Drug Delivery*.

[6] Chen D, Guo P, Chen S (2012). Properties of xyloglucan hydrogel as the biomedical sustained-release carriers. *Journal of Materials Science*.

[7] Uccello-Barretta G, Nazzi S, Zambito Y, Di Colo G, Balzano F, Sansò M (2010). Synergistic interaction between TS-polysaccharide and hyaluronic acid: implications in the formulation of eye drops. *International Journal of Pharmaceutics*.

[8] Takahashi A, Suzuki S, Kawasaki N (2002). Percutaneous absorption of non-steroidal anti-inflammatory drugs from in situ gelling xyloglucan formulations in rats. *International Journal of Pharmaceutics*.

[9] Mahajan HS, Tyagi V, Lohiya G, Nerkar P (2012). Thermally reversible xyloglucan gels as vehicles for nasal drug delivery. *Drug Delivery*.

[10] Strickland FM, Sun Y, Darvill A, Eberhard S, Pauly M, Albersheim P (2001). Preservation of the delayed-type hypersensitivity response to alloantigen by xyloglucans or oligogalacturonide does not correlate with the capacity to reject ultraviolet-induced skin tumors in mice. *The Journal of Investigative Dermatology*.

[11] Aravind SR, Joseph MM, Varghese S, Balaram P, Sreelekha TT (2012). Antitumor and immunopotentiating activity of polysaccharide PST001 isolated from the seed kernel of *Tamarindus indica*: an *in vivo* study in mice. *The Scientific World Journal*.

[12] do Rosário MMT, Kangussu-Marcolino MM, do Amaral AE, Noleto GR, Petkowicz CLDO (2011). Storage xyloglucans: potent macrophages activators. *Chemico-Biological Interactions*.

[13] Komutarin T, Azadi S, Butterworth L (2004). Extract of the seed coat of *Tamarindus indica* inhibits nitric oxide production by murine macrophages *in vitro* and *in vivo*. *Food and Chemical Toxicology*.

[14] Burgalassi S, Raimondi L, Pirisino R, Banchelli G, Boldrini E, Saettone MF (2000). Effect of xyloglucan (tamarind seed polysaccharide) on conjunctival cell adhesion to laminin and on corneal epithelium wound healing. *European Journal of Ophthalmology*.

[15] Rolando M, Valente C (2007). Establishing the tolerability and performance of tamarind seed polysaccharide (TSP) in treating dry eye syndrome: results of a clinical study. *BMC Ophthalmology*.

[16] Bin Mohamad MY, Akram HB, Bero DN, Rahman MT (2012). Tamarind seed extract enhances epidermal wound healing. *International Journal of Biology*.

[17] Kuchel JM, Barnetson RSC, Zhuang L, Strickland PM, Pelley RP, Halliday GM (2005). Tamarind inhibits solar-simulated ultraviolet radiation-induced suppression of recall responses in humans. *Letters in Drug Design & Discovery*.

[18] Hunt TK (1988). The physiology of wound healing. *Annals of Emergency Medicine*.

[19] Mutschler W (2012). Physiology and pathophysiology of wound healing of wound defects. *Der Unfallchirurg*.

[20] Maas M, Kemper M, Lamerding F, Klenke A, Deters A (2006). Variations in extraction protocol lead to differences in monosaccharide composition and bioactivity on human keratinocytes as shown by polysaccharides from banana and plum fruits. *Planta Medica*.

[21] Junchen C, Pufu L, Hengsheng S, Hengguang Z, Rutao F (2013). Effect of extraction methods on polysaccharide of clitocybe maxima stipe. *Advance Journal of Food Science and Technology*.

[22] Srivastava HC, Singh PP (1967). Structure of the polysaccharide from tamarind kernel. *Carbohydrate Research*.

[23] Deters AM, Schröder KR, Smiatek T, Hensel A (2005). Ispaghula (*Plantago ovata*) seed husk polysaccharides promote proliferation of human epithelial cells (skin keratinocytes and fibroblasts) via enhanced growth factor receptors and energy production. *Planta Medica*.

[24] Monsigny M, Petit C, Roche A-C (1988). Colorimetric determination of neutral sugars by a resorcinol sulfuric acid micromethod. *Analytical Biochemistry*.

[25] Bradford MM (1976). A rapid and sensitive method for the quantitation of microgram quantities of protein utilizing the principle of protein dye binding. *Analytical Biochemistry*.

[26] Abakuks S, Deters AM (2012). Polysaccharides of St. John's Wort herb stimulate NHDF proliferation and NEHK differentiation via influence on extracellular structures and signal pathways. *Advances in Pharmacology Science*.

[27] de Belder AN, Granath K (1973). Preparation and properties of fluorescein-labelled dextrans. *Carbohydrate Research*.

[28] Gescher K, Deters AM (2011). *Typha latifolia* L. fruit polysaccharides induce the differentiation and stimulate the proliferation of human keratinocytes *in vitro*. *Journal of Ethnopharmacology*.

[29] Mosmann T (1983). Rapid colorimetric assay for cellular growth and survival: application to proliferation and cytotoxicity assays. *Journal of Immunological Methods*.

[30] Schreier T, Degen E, Baschong W (1993). Fibroblast migration and proliferation during *in vitro* wound healing. A quantitative comparison between various growth factors and a low molecular weight blood dialyzate used in the clinic to normalize impaired wound healing. *Research in Experimental Medicine*.

[31] Maas M, Deters AM, Hensel A (2011). Anti-inflammatory activity of *Eupatorium perfoliatum* L. extracts, eupafolin, and dimeric guaianolide via iNOS inhibitory activity and modulation of inflammation-related cytokines and chemokines. *Journal of Ethnopharmacology*.

[32] The GeneCards Human, Crown Human Genome Center, Department of Molecular Genetics, Weizmann Institute of Science hostname: 356977-web1.xennexinc.com db genecards_309_100 index build: 100 solr: 1.4.

[33] Yang IV, Chen E, Hasseman JP (2002). Within the fold: assessing differential expression measures and reproducibility in microarray assays. *Genome Biology*.

[34] Berridge MV, Herst PM, Tan AS (2005). Tetrazolium dyes as tools in cell biology: new insights into their cellular reduction. *Biotechnology Annual Review*.

[35] Lang P, Kajiwara K (1993). Investigations of the architecture of tamarind seed polysaccharide in aqueous solution by different scattering techniques. *Journal of Biomaterials Science. Polymer Edition*.

[36] Gidley MJ, Lillford PJ, Rowlands DW (1991). Structure and solution properties of tamarind-seed polysaccharide. *Carbohydrate Research*.

[37] Li Y, Corradetti MN, Inoki K, Guan K-L (2004). TSC2: filling the GAP in the mTOR signaling pathway. *Trends in Biochemical Sciences*.

[38] Zhou J, Wulfkuhle J, Zhang H (2007). Activation of the PTEN/mTOR/STAT3 pathway in breast cancer stem-like cells is required for viability and maintenance. *Proceedings of the National Academy of Sciences of the United States of America*.

[39] Ricardo-Gonzalez RR, Eagle AR, Odegaard JI (2010). IL-4/STAT6 immune axis regulates peripheral nutrient metabolism and insulin sensitivity. *Proceedings of the National Academy of Sciences of the United States of America*.

[40] Roux PP, Blenis J (2004). ERK and p38 MAPK-activated protein kinases: a family of protein kinases with diverse biological functions. *Microbiology and Molecular Biology Reviews*.

[41] Gallo KA, Johnson GL (2002). Mixed-lineage kinase control of JNK and p38 MAPK pathways. *Nature Reviews Molecular Cell Biology*.

[42] Andersson ER (2012). The role of endocytosis in activating and regulating signal transduction. *Cellular and Molecular Life Sciences*.

[43] Ilangumaran S, Borisch B, Hoessli DC (1999). Signal transduction via CD44: role of plasma membrane microdomains. *Leukemia & Lymphoma*.

[44] Krauss M, Haucke V (2011). Shaping membranes for endocytosis. *Reviews of Physiology, Biochemistry and Pharmacology*.

[45] Bienz M (2005). *β*-catenin: a pivot between cell adhesion and Wnt signalling. *Current Biology*.

[46] Weston CR, Davis RJ (2002). The JNK signal transduction pathway. *Current Opinion in Genetics and Development*.

[47] Sano M, Miyata E, Tamano S, Hagiwara A, Ito N, Shirai T (1996). Lack of carcinogenicity of tamarind seed polysaccharide in B6C3F1 mice. *Food and Chemical Toxicology*.

